# Supplementary material for: Age-dependent heterogeneity in the antigenic effects of mutations to influenza hemagglutinin
Source: bioRxiv. 2023 Dec 12:2023.12.12.571235. Preprint. [Version 1] doi: 10.1101/2023.12.12.571235 (PMC10760046; doi:10.1101/2023.12.12.571235)
Supplement: Supplement 1 [file NIHPP2023.12.12.571235V1-supplement-1.pdf]

bioRxiv preprint doi: https://doi.org/10.1101/2023.12.12.571235; this version posted December 12, 2023. The copyright holder for this preprint (which was not certified by peer review) is the author/funder, who has granted bioRxiv a license to display the preprint in perpetuity. It is made available under aCC-BY 4.0 International license.

Figure S1

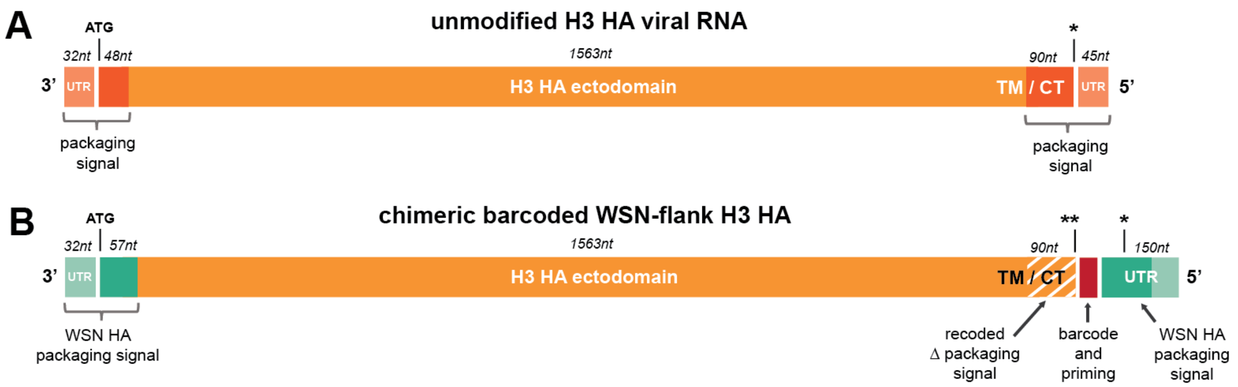

Figure S1. Design of chimeric barcoded WSN-flank H3 HA construct. All segments are shown in the reverse orientation of the negative-sense viral genome, with the 3' to 5' labels indicating ends in the negative-sense viral genome. Stop codons are denoted as asterisks. (A) Schematic of normal unmodified H3 HA. Influenza is multi-segmented, and proper packaging of vRNA into the virion relies on segment-specific RNA sequences called "packaging signals." These span both coding and noncoding regions at the 3' and 5' ends of the vRNA segment (Li et al., 2021). Packaging signals, untranslated regions (UTRs), ectodomain, transmembrane domain (TM), and cytoplasmic tail (CT) are labeled for a normal H3 HA. Corresponding nucleotide length is labeled based on sequence of A/Hong Kong/45/2019. (B) Schematic of chimeric WSN-flank H3 HA used for barcoding. To insert a barcode without disrupting vRNA packaging, we duplicate the full 5' packaging signal (including sequence from the coding region), and place it after the stop codons of the HA gene. The second stop codon was introduced to minimize polymerase read-through. We then insert a 16nt barcode and constant priming sequence after the stop codon and before the intact packaging signal. The native packaging sequence in the 5' coding region is synonymously recoded to avoid interference between duplicated sequences. Homology between the native and duplicated packaging signal could lead to barcode loss; we therefore included a stop codon in the duplicated packaging signal that will be in-frame if it replaces the native packaging signal. This ensures that loss of the barcode will generate a truncated, non-functional HA protein. The 3' and duplicated 5' packaging signals are taken from the lab-adapted A/WSN/1933 HA, as the library is grown using the other seven gene segments from this strain, and consistency between packaging signals helps achieve higher titers. See https://github.com/dms-vep/flu_h3_hk19_dms/tree/main/library_design/plasmid_maps for an annotated plasmid map of WSN-flank H3 HA.

bioRxiv preprint doi: https://doi.org/10.1101/2023.12.12.571235; this version posted December 12, 2023. The copyright holder for this preprint (which was not certified by peer review) is the author/funder, who has granted bioRxiv a license to display the preprint in perpetuity. It is made available under aCC-BY 4.0 International license.

Figure S2

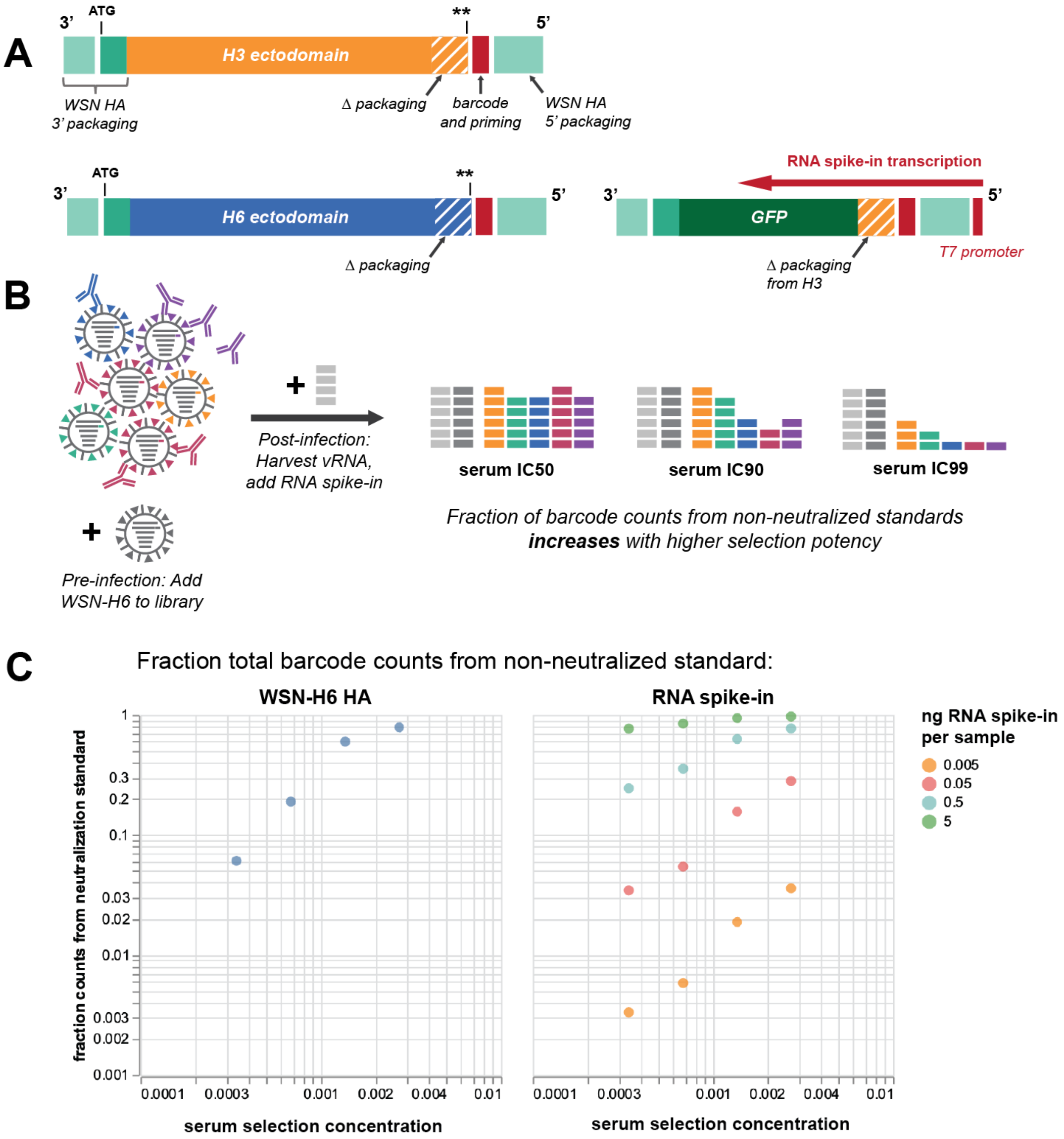

Figure S2. Incorporation of non-neutralized standards to generate quantitative escape measurements. (A) Design of WSN-H6 HA and RNA spike-in neutralization standards. The WSN-H6 HA standard is identical to the H3 HA library construct, but uses the ectodomain and 5' region from A/Turkey/Mass/1975, a low-pathogenicity avian HA strain (GenBank Accession AB296072.1) (Sandbulte et al., 2009). The RNA spike-in is generated by *in vitro* transcription of a construct where the H3 HA ectodomain is replaced by GFP. See https://github.com/dms-vep/flu_h3_hk19_dms/tree/main/library_design/plasmid_maps for

bioRxiv preprint doi: https://doi.org/10.1101/2023.12.12.571235; this version posted December 12, 2023. The copyright holder for this preprint (which was not certified by peer review) is the author/funder, who has granted bioRxiv a license to display the preprint in perpetuity. It is made available under aCC-BY 4.0 International license.

annotated plasmid maps of both standards. (B) WSN-H6 HA is spiked into the H3 HA library before incubating with serum and infecting cells. The RNA spike-in standard is added when harvesting cellular RNA at 13 hours post-infection. As neither standard is neutralized by human sera, the number of barcodes from the standards remains constant, while H3 HA barcodes decrease at higher concentrations of neutralizing antibodies. The overall fraction of standard barcodes should therefore increase at a constant rate with increasing serum potency. (C) At a range of serum concentrations used for selections, the fraction of counts from the WSN-H6 HA neutralization standard increases in a consistent manner as the H3 HA variant library is more potently neutralized. Spiking in 0.05ng of RNA standard per sample achieves the same effect.

bioRxiv preprint doi: https://doi.org/10.1101/2023.12.12.571235; this version posted December 12, 2023. The copyright holder for this preprint (which was not certified by peer review) is the author/funder, who has granted bioRxiv a license to display the preprint in perpetuity. It is made available under aCC-BY 4.0 International license.

Figure S3

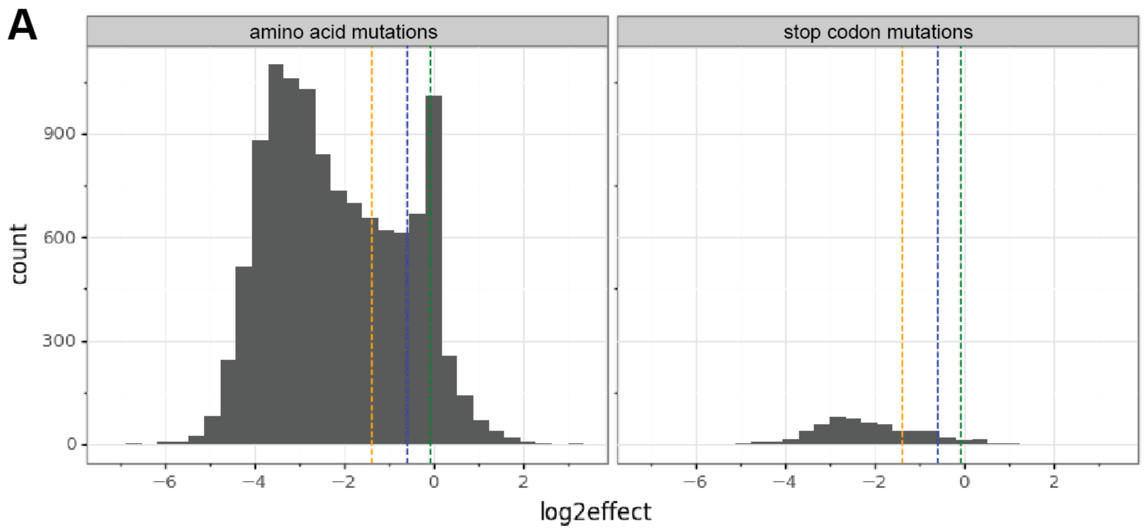

B recently mutated epitopes:

A	B	E	other
128	159	62	121
131	160	83	171
135	186	92	311
138	190	261	326
142	192		
144	193		
	197		
	198		

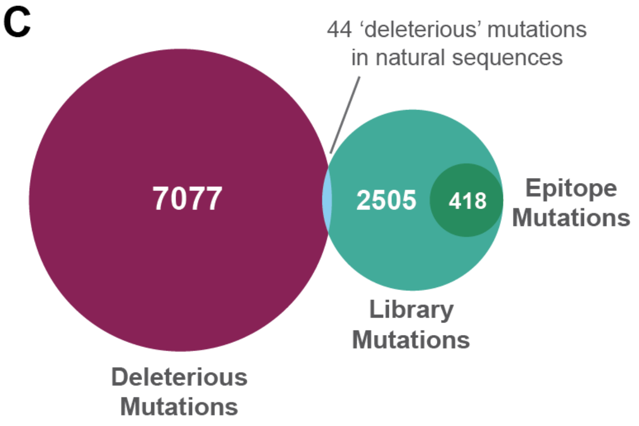

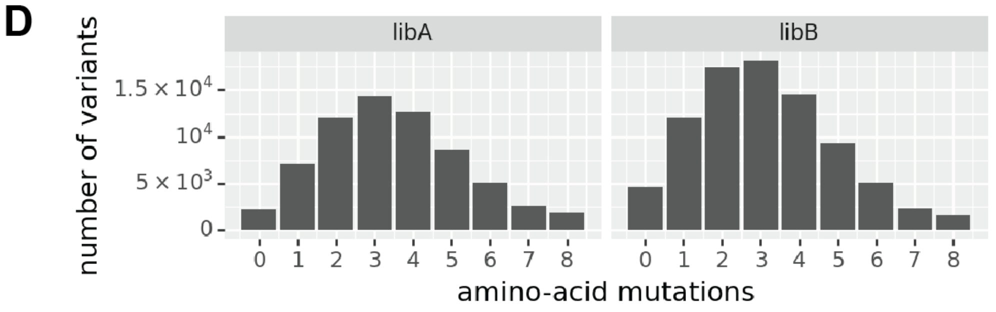

Figure S3. Composition of H3 HA libraries. (A) Functional effects of all single amino acid mutations to A/Perth/16/2009 H3 HA, calculated by Lee et al. (2018). Vertical lines delimit 75%, 90%, and 95% of the most deleterious stop codons. These quantiles are transposed onto all amino acid mutations, and 7,077 mutants corresponding to the 75th quantile of stop codons were excluded from the current library. (B) List of epitope sites, defined as sites that have been mutated in at least one major H3 HA clade between 2015 and 2021. These sites are randomly mutagenized in the library at a higher rate than the general non-deleterious mutations. (C) Visualization of

bioRxiv preprint doi: https://doi.org/10.1101/2023.12.12.571235; this version posted December 12, 2023. The copyright holder for this preprint (which was not certified by peer review) is the author/funder, who has granted bioRxiv a license to display the preprint in perpetuity. It is made available under aCC-BY 4.0 International license.

types of mutations included in the library. In total, 7,077 mutations in the H3 HA ectodomain region were identified as functionally deleterious. 44 of these excluded mutants were identified in H3 HA strains circulating between 1968 and 2021, and added back into the library. This left 2,505 mutations to target in the library. 418 mutations at epitope sites were introduced at a higher frequency than general library mutations. (D) Distribution of mutations per variant in two fully independent plasmid libraries.

bioRxiv preprint doi: https://doi.org/10.1101/2023.12.12.571235; this version posted December 12, 2023. The copyright holder for this preprint (which was not certified by peer review) is the author/funder, who has granted bioRxiv a license to display the preprint in perpetuity. It is made available under aCC-BY 4.0 International license.

Figure S4

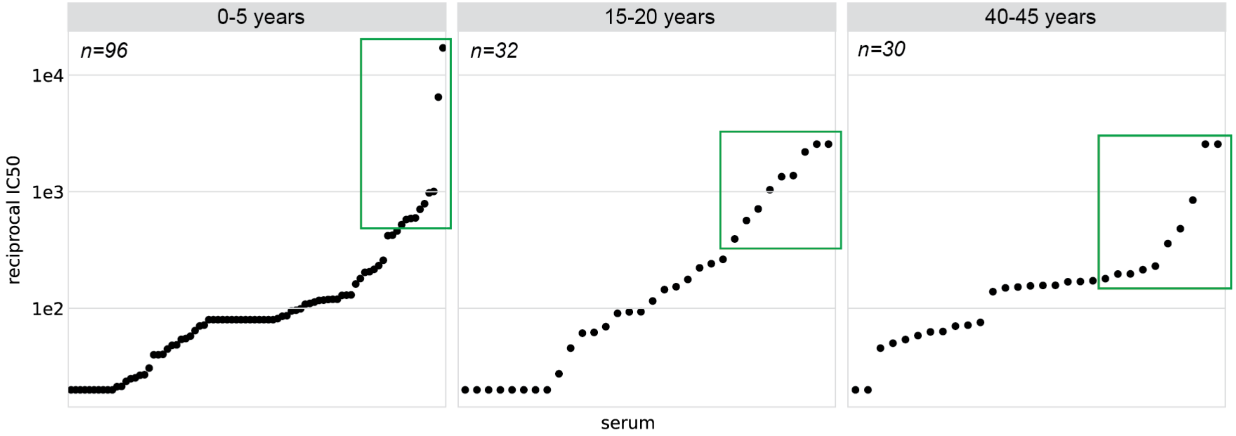

Figure S4. Serum neutralizing titers against the A/Hong Kong/45/2019 HA barcoded library strain. Neutralization assays were performed using influenza carrying GFP in the PB1 segment, as described previously (Doud et al., 2018; Hooper and Bloom, 2013; see https://github.com/jbloomlab/flu_PB1flank-GFP_neut_assay for detailed protocol). Only a single replicate was run for each serum. IC50 values were calculated by fitting Hill-like curves using the neutcurve Python package (https://jbloomlab.github.io/neutcurve/). The ten sera from each age group with the highest neutralization activity against the parental library strain, boxed in green, were selected for serum escape mapping.

bioRxiv preprint doi: https://doi.org/10.1101/2023.12.12.571235; this version posted December 12, 2023. The copyright holder for this preprint (which was not certified by peer review) is the author/funder, who has granted bioRxiv a license to display the preprint in perpetuity. It is made available under aCC-BY 4.0 International license.

Figure S5

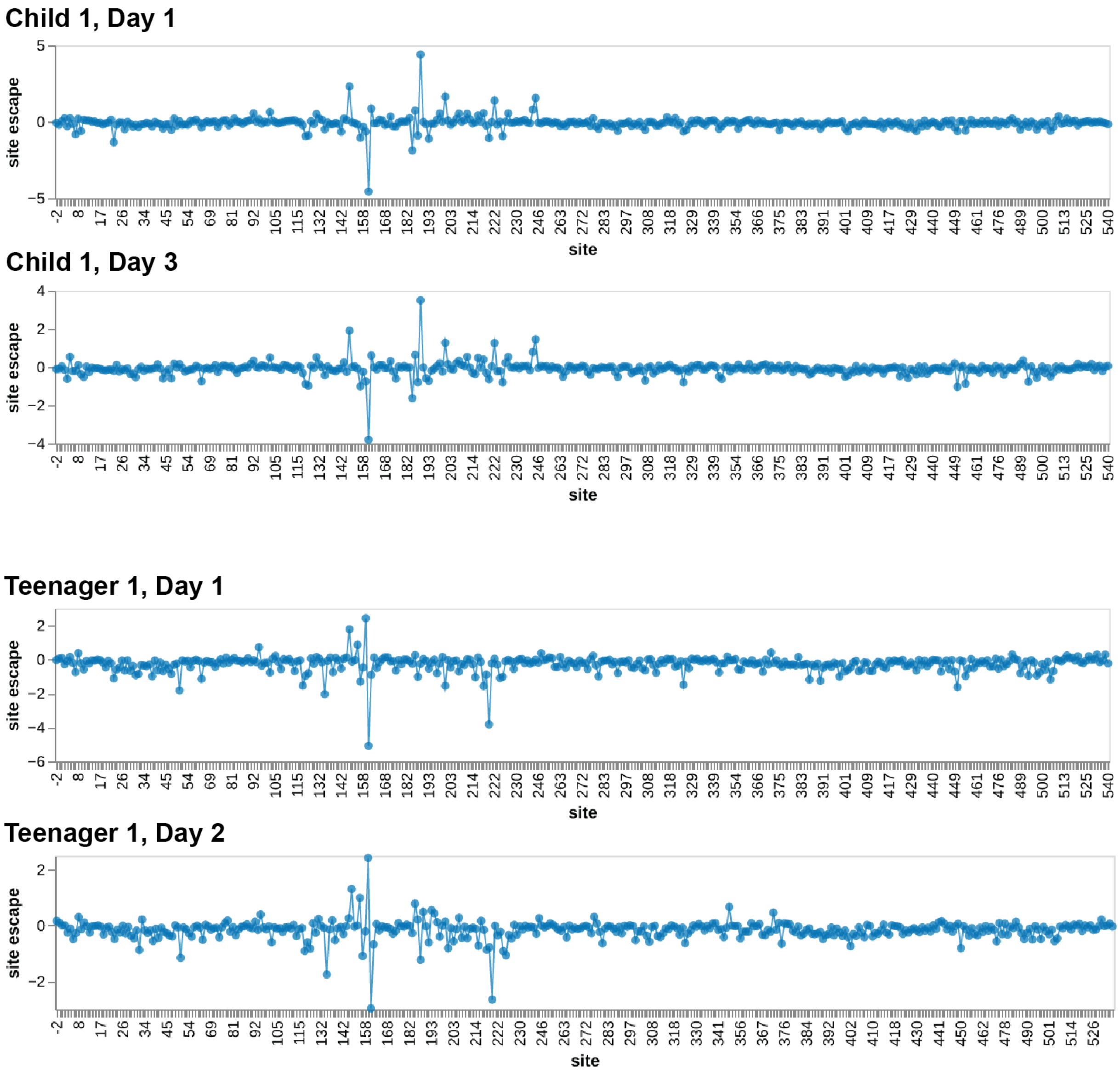

Figure S5. Escape maps for sera collected on different days from the same individual. One set of repeated samples comes from a child (sample 2388), and the other from a teenager (sample 3862). Line plots show summed escape scores of each sampled mutation at that site.

bioRxiv preprint doi: https://doi.org/10.1101/2023.12.12.571235; this version posted December 12, 2023. The copyright holder for this preprint (which was not certified by peer review) is the author/funder, who has granted bioRxiv a license to display the preprint in perpetuity. It is made available under aCC-BY 4.0 International license.

Figure S6

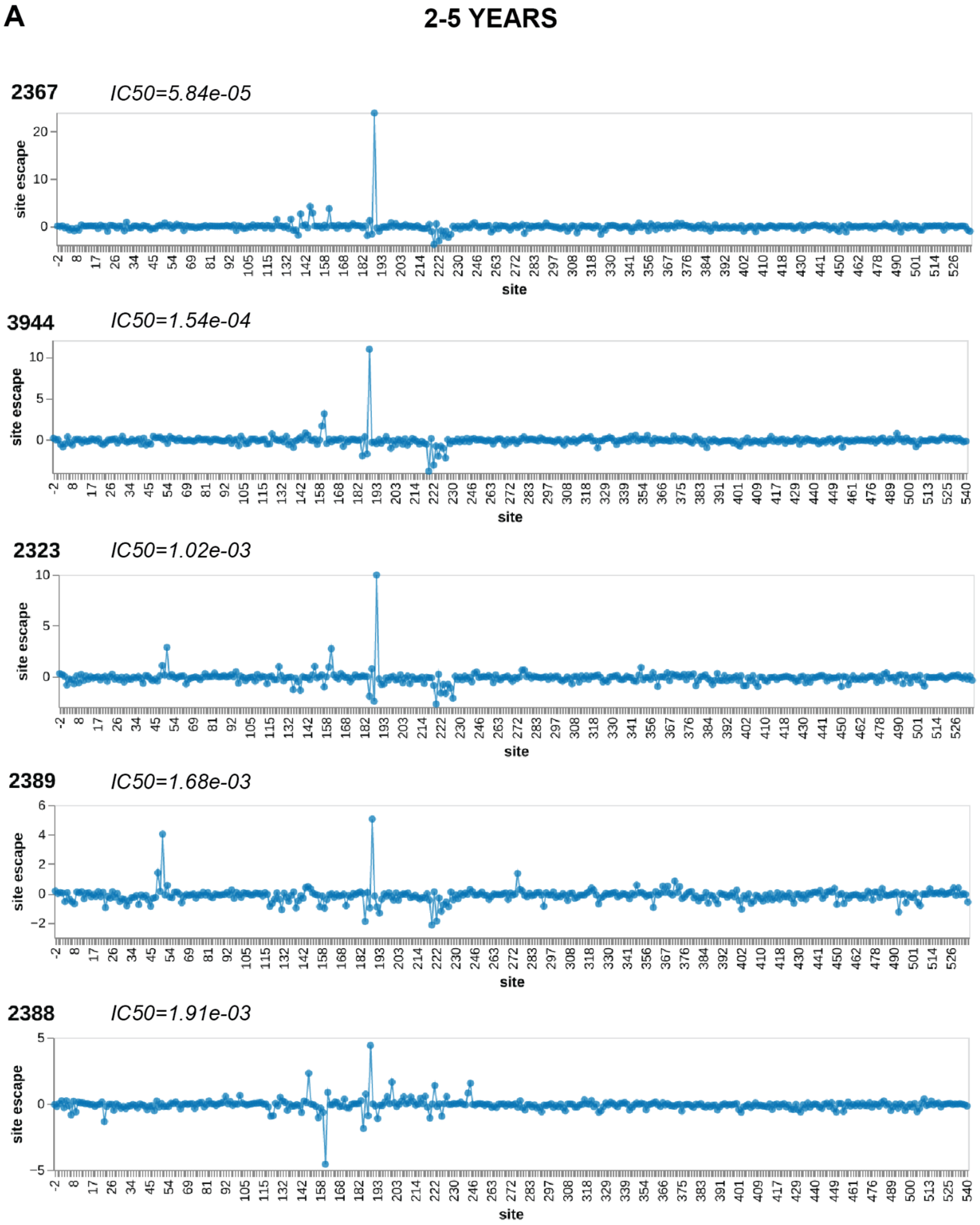

bioRxiv preprint doi: https://doi.org/10.1101/2023.12.12.571235; this version posted December 12, 2023. The copyright holder for this preprint (which was not certified by peer review) is the author/funder, who has granted bioRxiv a license to display the preprint in perpetuity. It is made available under aCC-BY 4.0 International license.

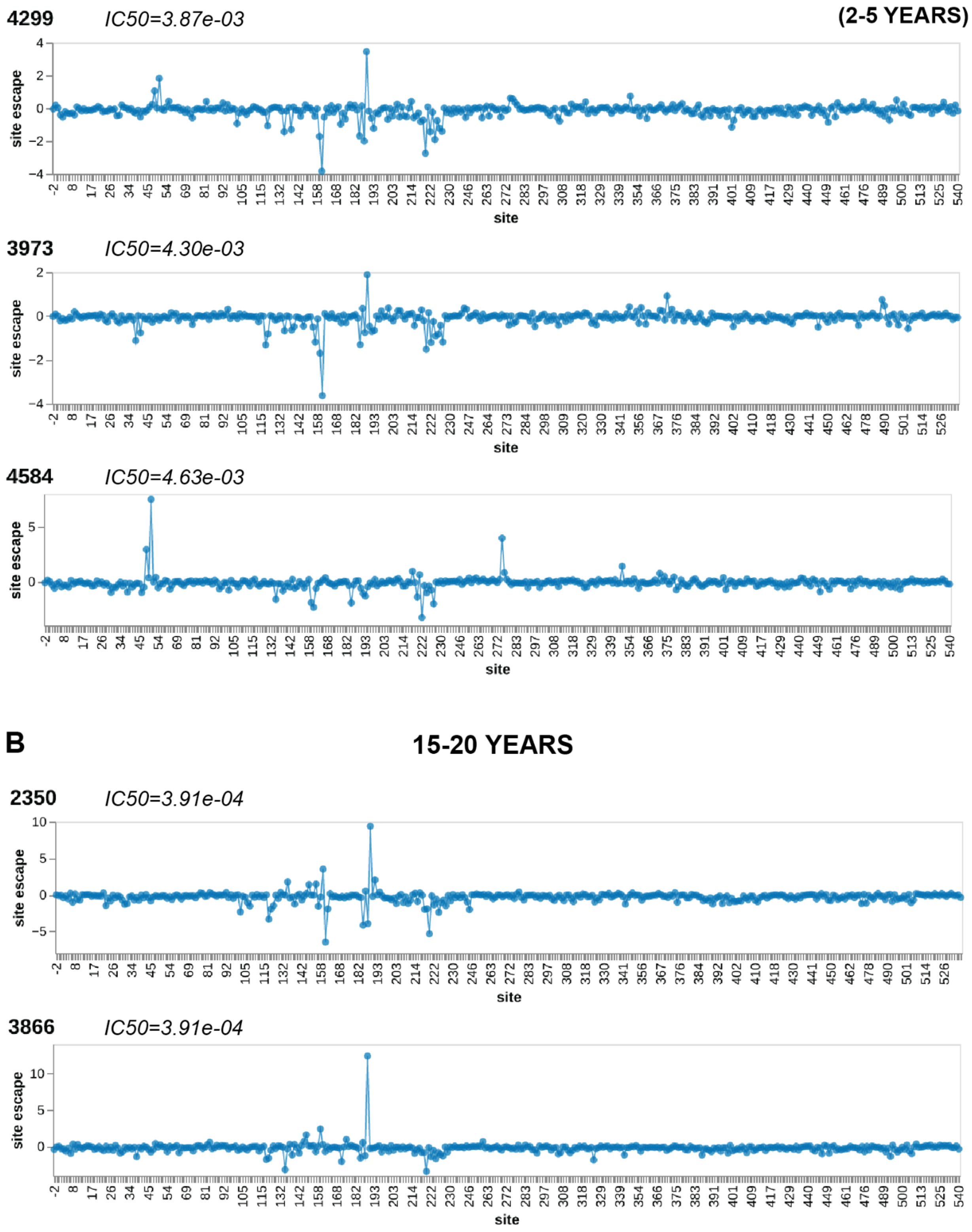

bioRxiv preprint doi: https://doi.org/10.1101/2023.12.12.571235; this version posted December 12, 2023. The copyright holder for this preprint (which was not certified by peer review) is the author/funder, who has granted bioRxiv a license to display the preprint in perpetuity. It is made available under aCC-BY 4.0 International license.

(15-20 YEARS)

2365 *IC50=4.56e-04*

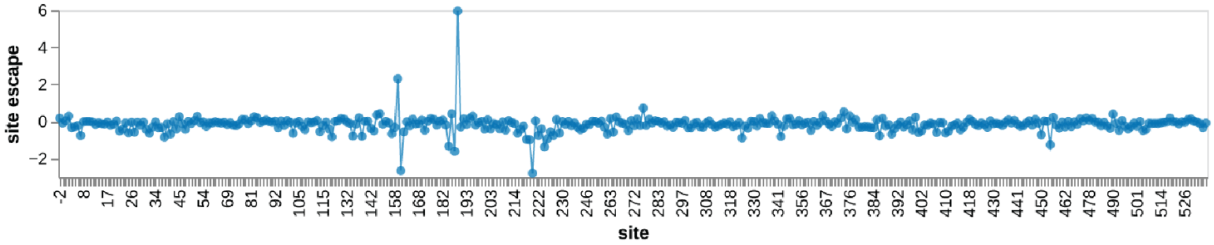

2382 *IC50=7.43e-04*

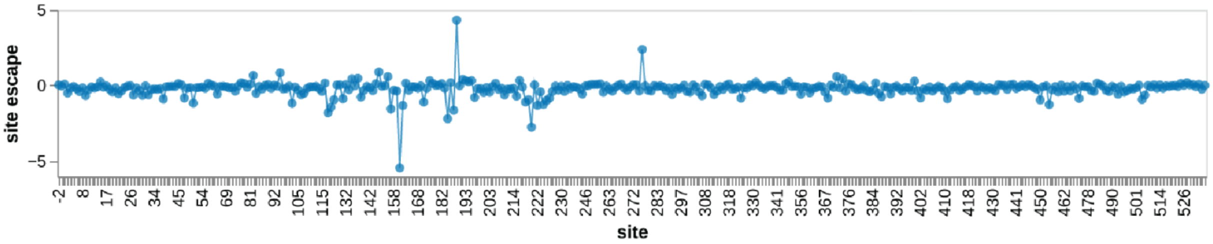

3862 *IC50=9.61e-04*

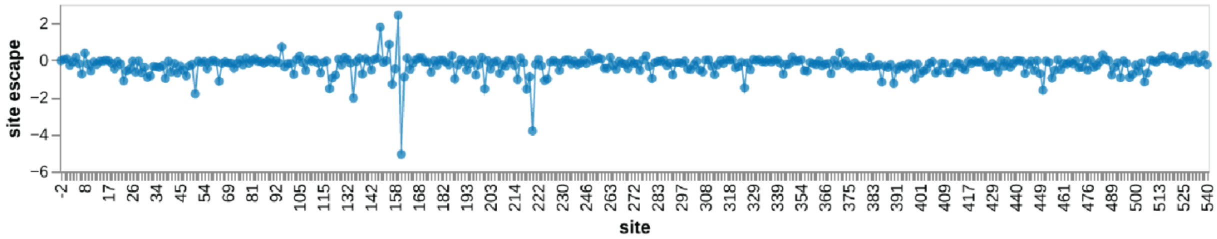

3857 *IC50=2.54e-03*

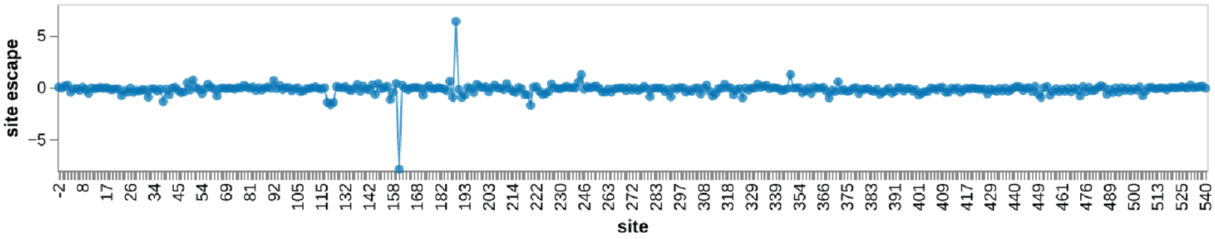

2380 *IC50=3.80e-03*

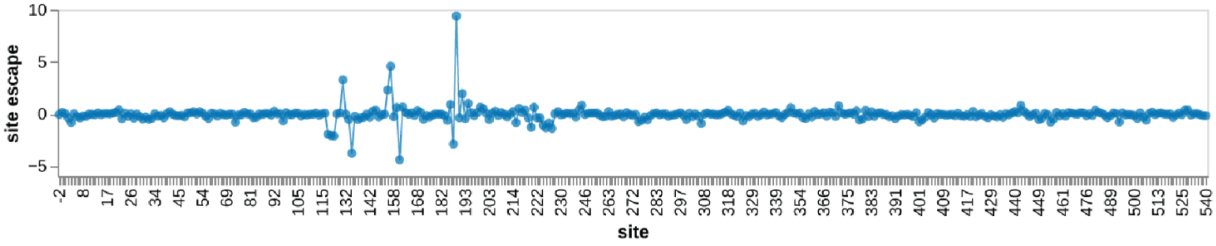

bioRxiv preprint doi: https://doi.org/10.1101/2023.12.12.571235; this version posted December 12, 2023. The copyright holder for this preprint (which was not certified by peer review) is the author/funder, who has granted bioRxiv a license to display the preprint in perpetuity. It is made available under aCC-BY 4.0 International license.

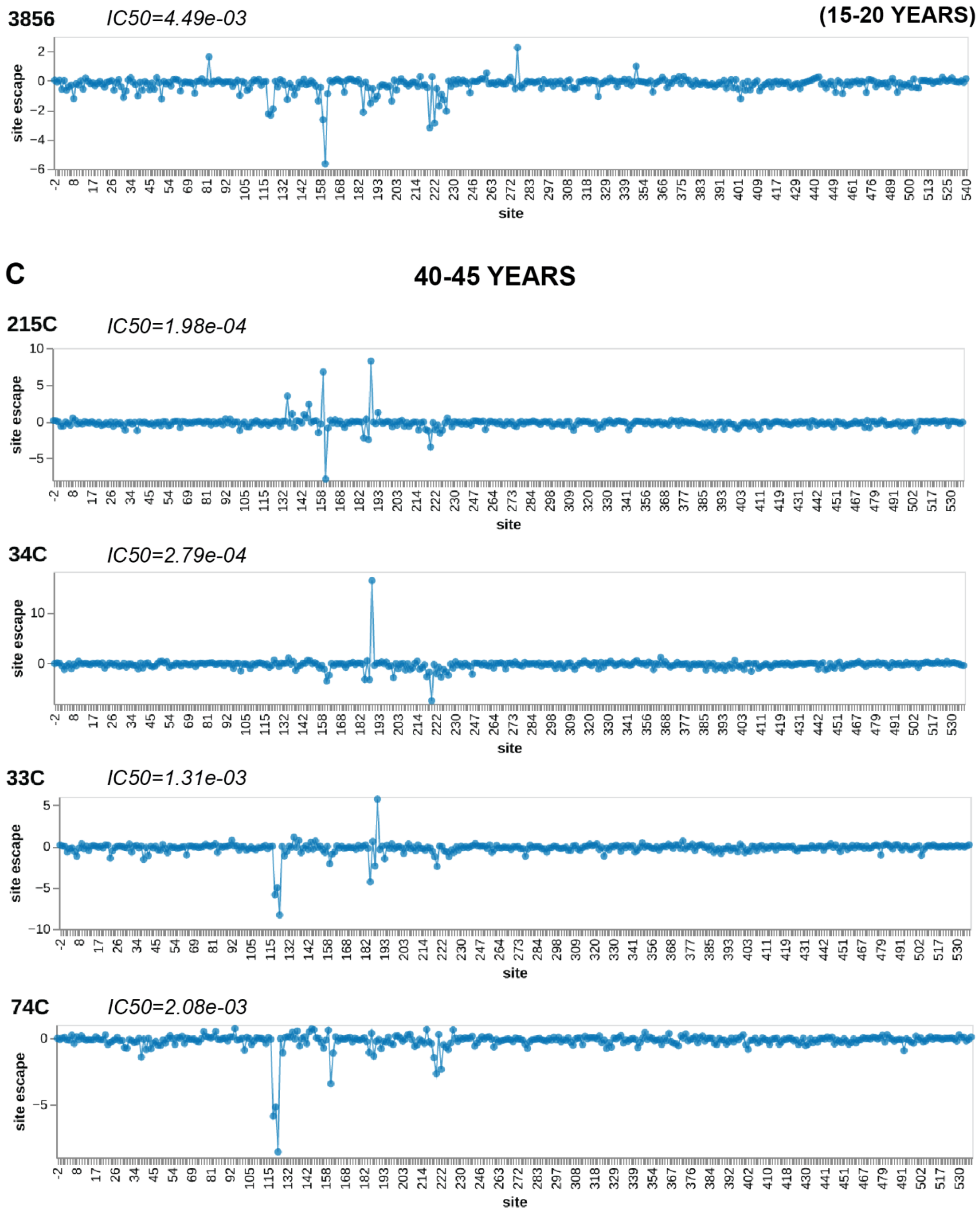

bioRxiv preprint doi: https://doi.org/10.1101/2023.12.12.571235; this version posted December 12, 2023. The copyright holder for this preprint (which was not certified by peer review) is the author/funder, who has granted bioRxiv a license to display the preprint in perpetuity. It is made available under aCC-BY 4.0 International license.

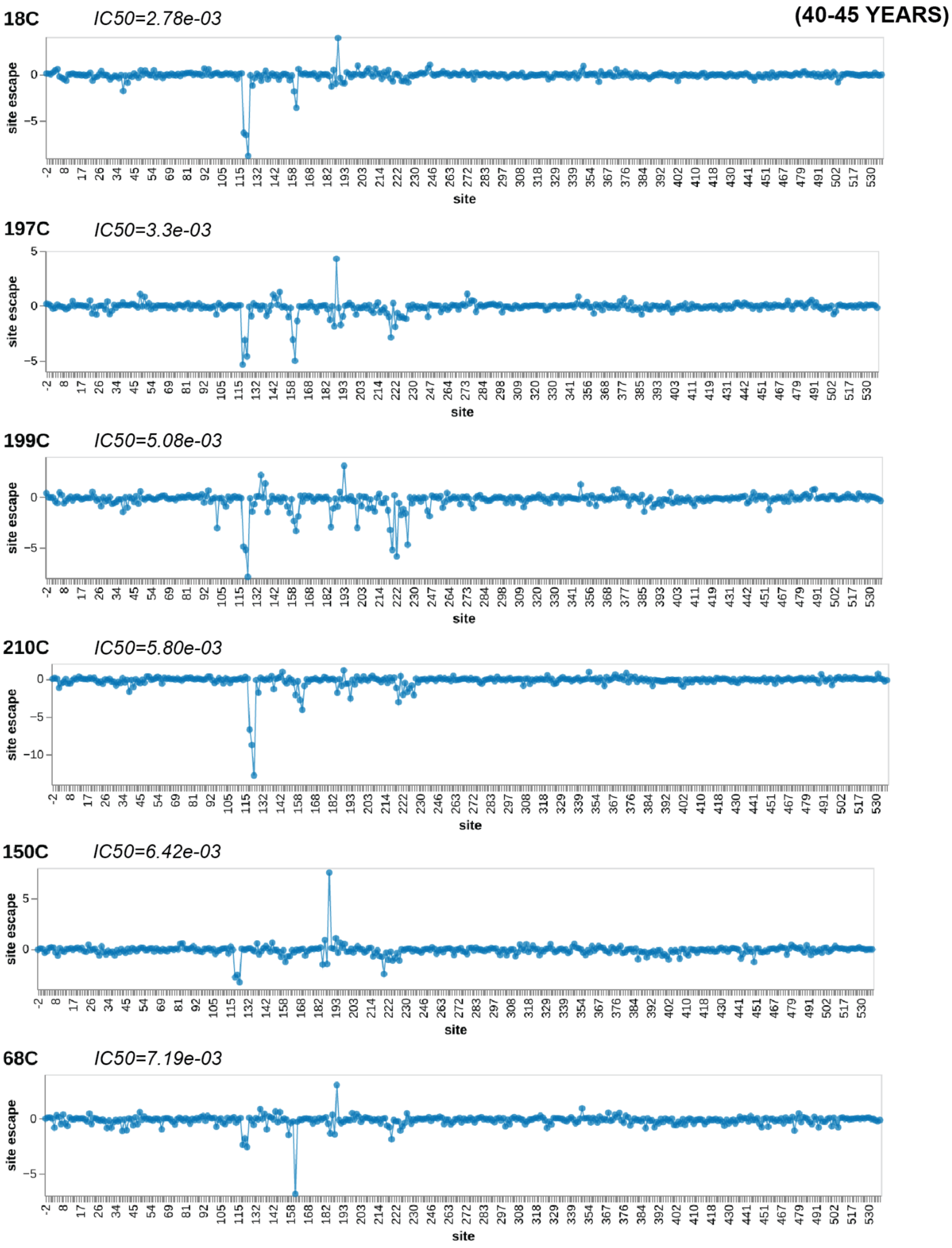

bioRxiv preprint doi: https://doi.org/10.1101/2023.12.12.571235; this version posted December 12, 2023. The copyright holder for this preprint (which was not certified by peer review) is the author/funder, who has granted bioRxiv a license to display the preprint in perpetuity. It is made available under aCC-BY 4.0 International license.

D **68 YEARS**

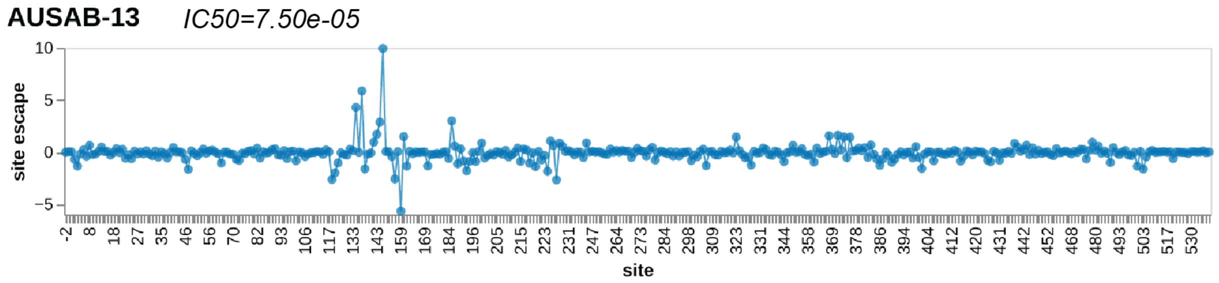

E **INFANT (2 months)**

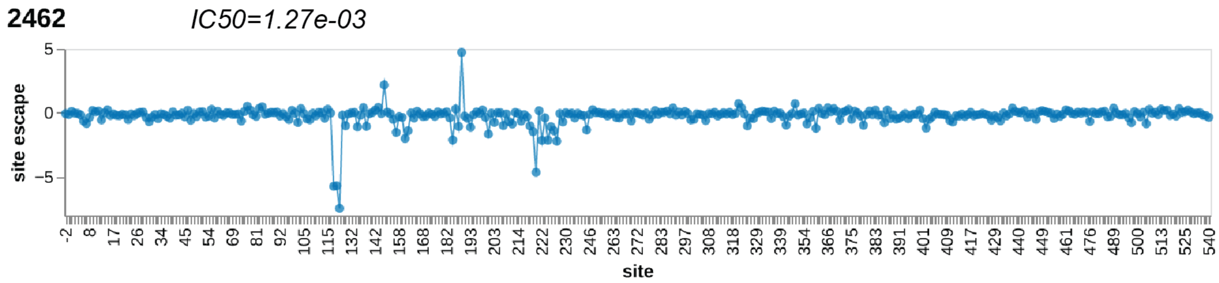

Figure S6. Individual escape maps for each serum in the 2020 cohort, analyzed against the A/Hong Kong/45/2019 library. Line plots show summed escape scores of each sampled mutation at that site. Sera are ordered by decreasing potency within each age group, with IC50s labeled for each serum. (A) Child sera, (B) teenage sera, (C) adult sera, (D) elderly serum, and (E) infant serum.

bioRxiv preprint doi: https://doi.org/10.1101/2023.12.12.571235; this version posted December 12, 2023. The copyright holder for this preprint (which was not certified by peer review) is the author/funder, who has granted bioRxiv a license to display the preprint in perpetuity. It is made available under aCC-BY 4.0 International license.

Figure S7

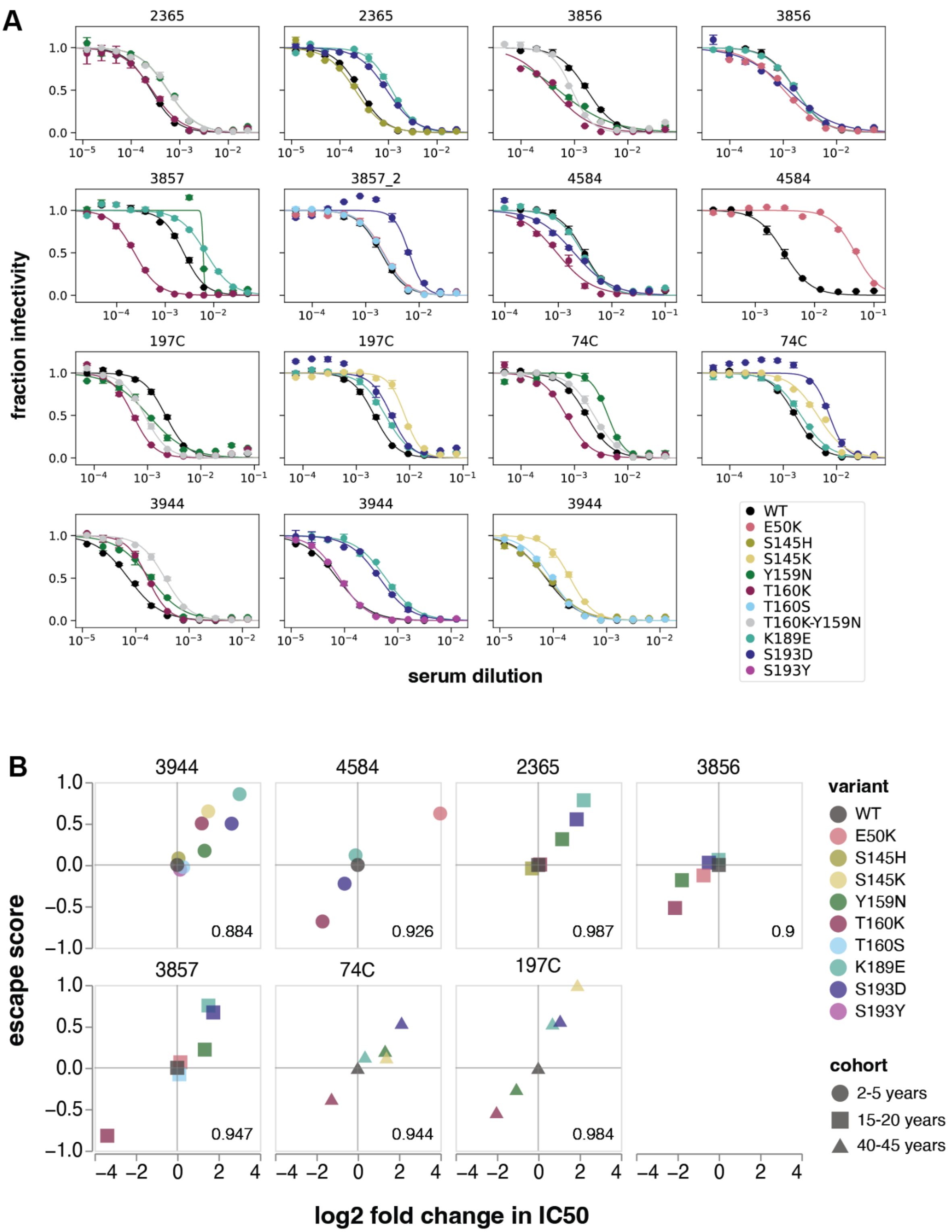

Figure S7. Neutralization assay results for validation of deep mutational scanning measurements against the A/Hong Kong/45/2019 library. (A) Neutralization curves for unmutated A/Hong Kong/45/2019 H3 HA ('WT') and selected mutants against seven representative

bioRxiv preprint doi: https://doi.org/10.1101/2023.12.12.571235; this version posted December 12, 2023. The copyright holder for this preprint (which was not certified by peer review) is the author/funder, who has granted bioRxiv a license to display the preprint in perpetuity. It is made available under aCC-BY 4.0 International license.

sera from different age cohorts. (B) Correlation between escape score and the log2 fold change in serum IC50 for each variant, plotted for each serum independently. Pearson R correlation is noted on each plot. The IC50 for each variant was compared to the IC50 for the wildtype strain run in the same experiment, to control for potential variation in serum concentration and cell number between independent experiments.

bioRxiv preprint doi: https://doi.org/10.1101/2023.12.12.571235; this version posted December 12, 2023. The copyright holder for this preprint (which was not certified by peer review) is the author/funder, who has granted bioRxiv a license to display the preprint in perpetuity. It is made available under aCC-BY 4.0 International license.

Figure S8

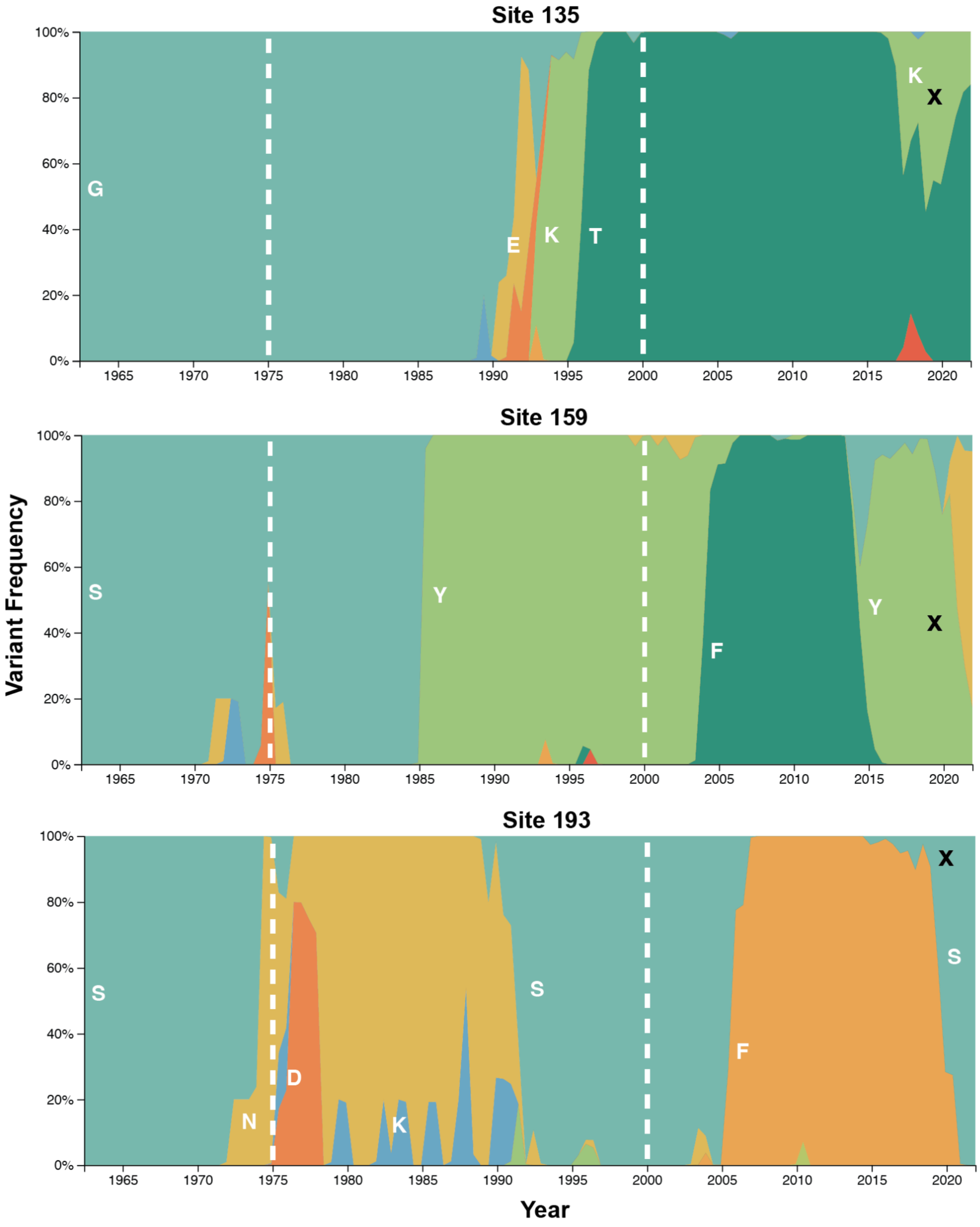

Figure S8. Global frequency of variants at sites 135, 159, and 193. Dashed lines indicate the earliest birthdate of individuals in the adult cohort (1975) and teenage cohort (2000). Black 'X' indicates the amino acid identity of the wildtype library strain, A/Hong Kong/45/2019. Frequency plot adapted from the Nextstrain real-time pathogen evolution website (Hadfield et al., 2018; Neher and Bedford, 2015).

bioRxiv preprint doi: https://doi.org/10.1101/2023.12.12.571235; this version posted December 12, 2023. The copyright holder for this preprint (which was not certified by peer review) is the author/funder, who has granted bioRxiv a license to display the preprint in perpetuity. It is made available under aCC-BY 4.0 International license.

Figure S9

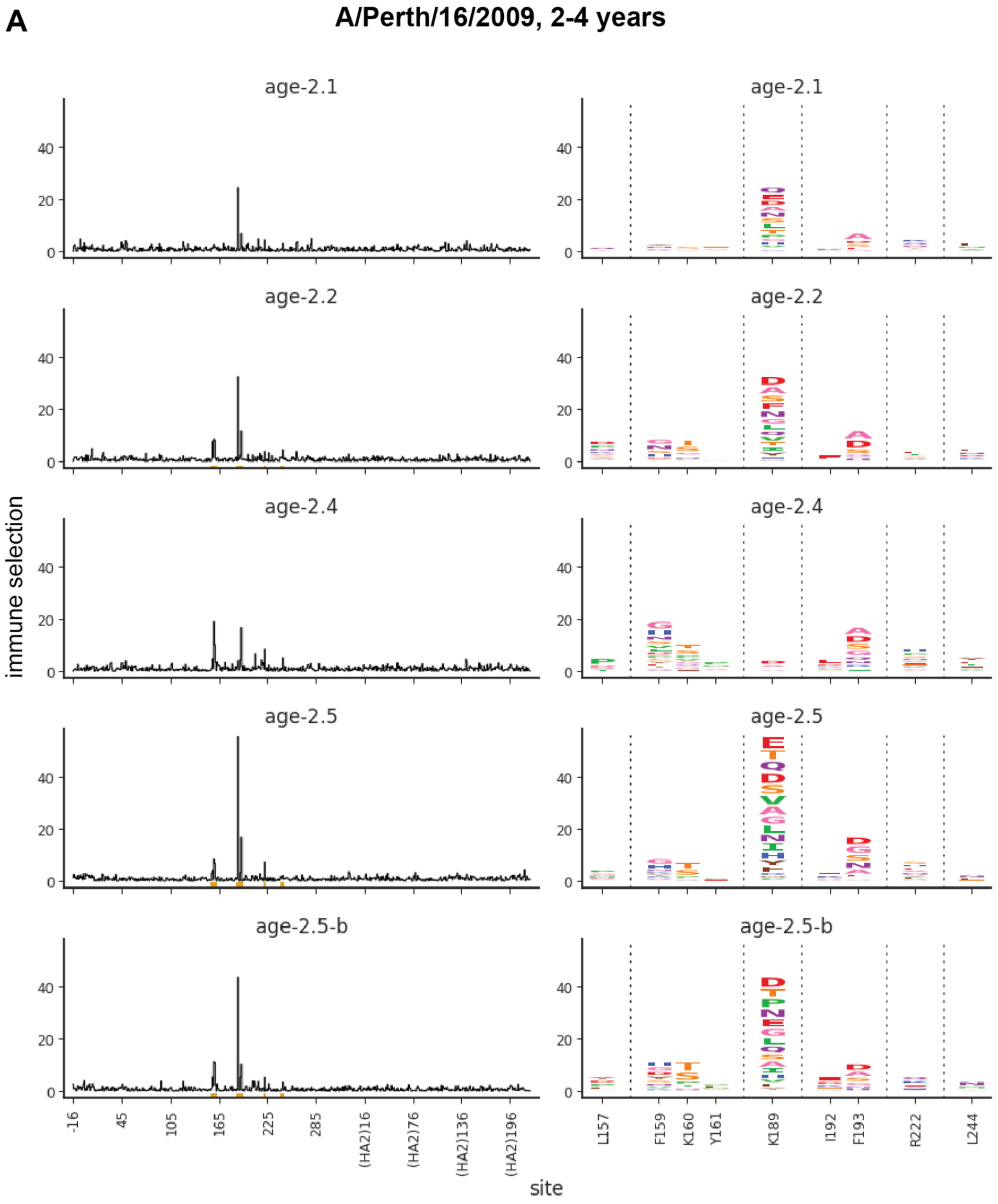

bioRxiv preprint doi: https://doi.org/10.1101/2023.12.12.571235; this version posted December 12, 2023. The copyright holder for this preprint (which was not certified by peer review) is the author/funder, who has granted bioRxiv a license to display the preprint in perpetuity. It is made available under aCC-BY 4.0 International license.

A/Perth/16/2009, 2-4 years (cont.)

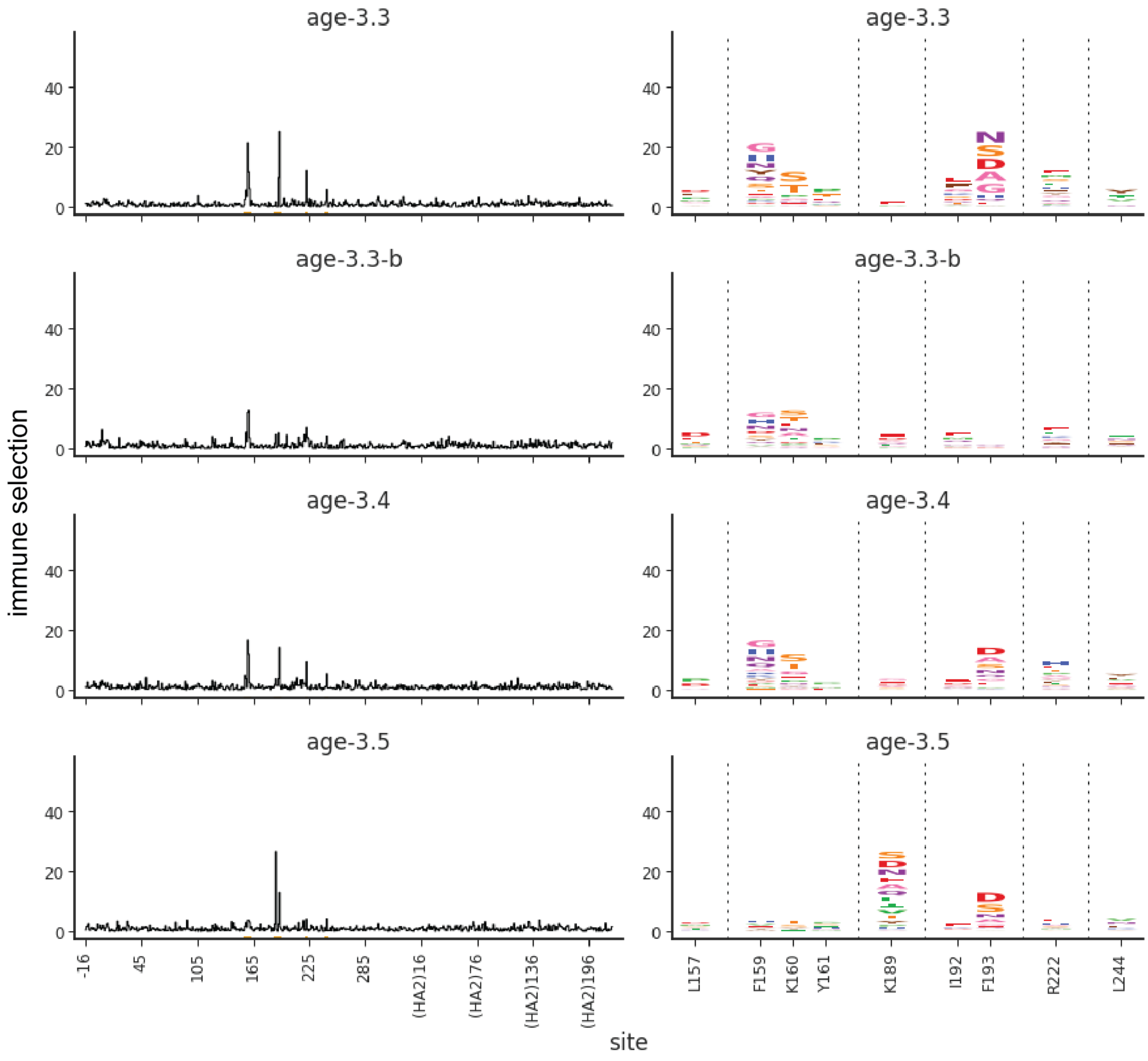

bioRxiv preprint doi: https://doi.org/10.1101/2023.12.12.571235; this version posted December 12, 2023. The copyright holder for this preprint (which was not certified by peer review) is the author/funder, who has granted bioRxiv a license to display the preprint in perpetuity. It is made available under aCC-BY 4.0 International license.

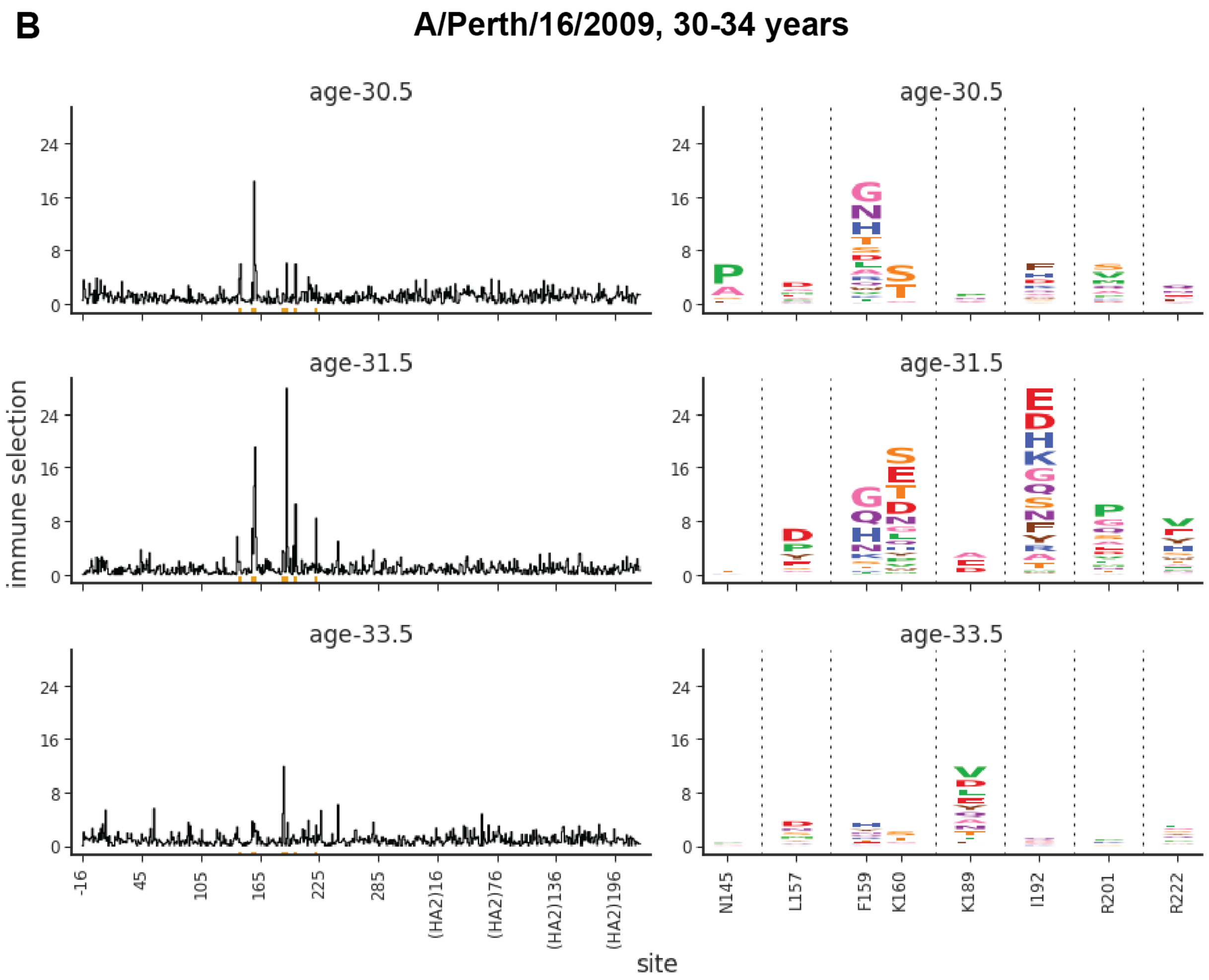

Figure S9. Individual escape maps for each serum in the 2010-2011 cohort, analyzed against the A/Perth/16/2009 library. Line plots show summed escape scores of each sampled mutation at that site. Logo plots show mutation-level escape at key sites, where the height of each letter corresponds to the escape score for that amino acid substitution. (A) Child sera, (B) adult sera.

bioRxiv preprint doi: https://doi.org/10.1101/2023.12.12.571235; this version posted December 12, 2023. The copyright holder for this preprint (which was not certified by peer review) is the author/funder, who has granted bioRxiv a license to display the preprint in perpetuity. It is made available under aCC-BY 4.0 International license.

Figure S10

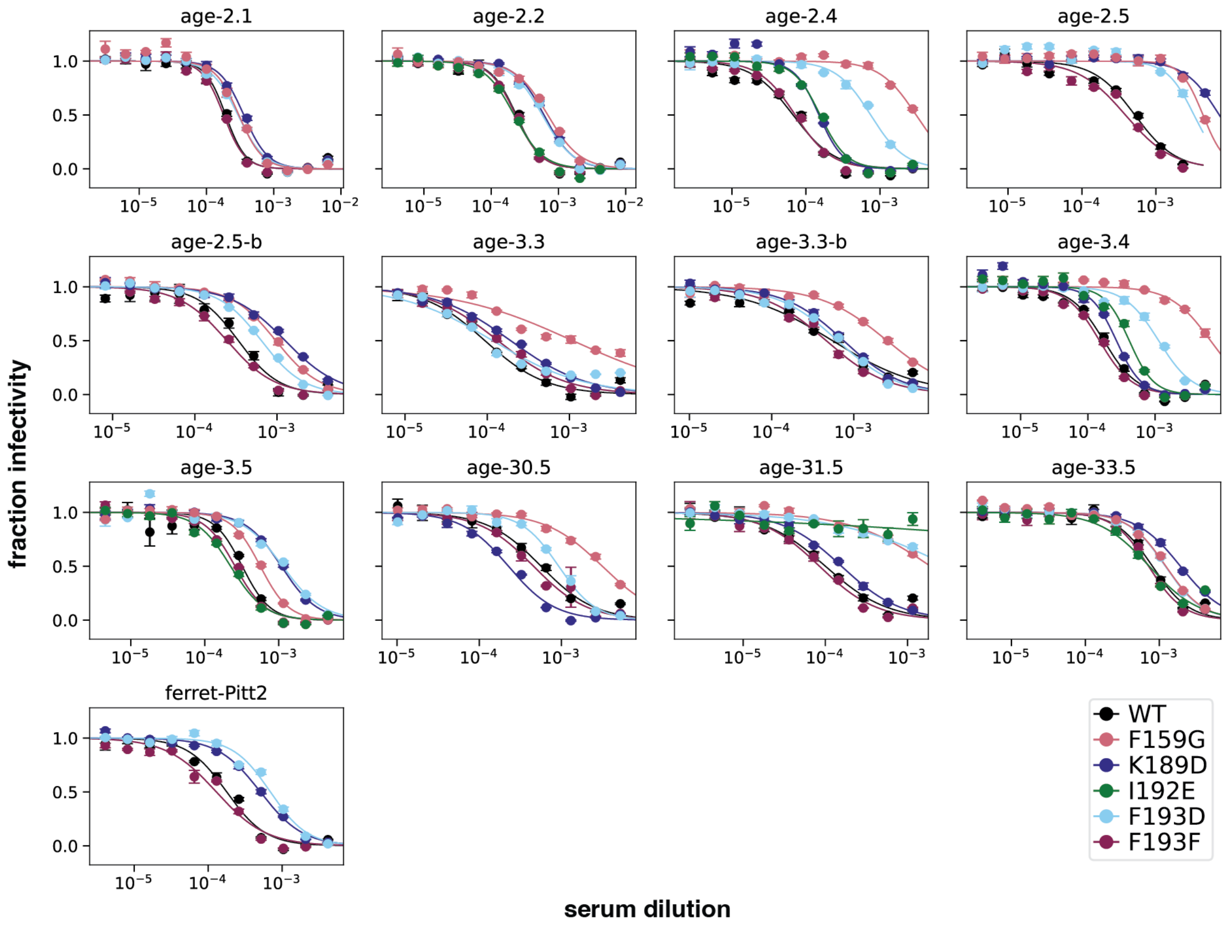

Figure S10. Neutralization assay results for validation of deep mutational scanning measurements against the A/Perth/16/2009 library. Neutralization curves are shown for unmutated A/Perth/16/2009 H3 HA ('WT') and selected mutants against all human sera from the 2010-2011 cohort, plus one representative ferret serum.

bioRxiv preprint doi: https://doi.org/10.1101/2023.12.12.571235; this version posted December 12, 2023. The copyright holder for this preprint (which was not certified by peer review) is the author/funder, who has granted bioRxiv a license to display the preprint in perpetuity. It is made available under aCC-BY 4.0 International license.

Table S1.

Study ID	Age at serum collection (in years)	Serum collection date (quarter)	Flu shot 1 date (quarter)	Flu shot 2 date (quarter)	Flu shot 3 date (quarter)	Immunocompromised	Recent intravenous IgG treatment?	Infection history
2462	0.2	Apr-June 2020	None	None	None	No	No	unknown
2323	3	Apr-June 2020	Oct-Dec 2019	Oct-Dec 2018	Oct-Dec 2017	No	No	unknown
2367	3	Apr-June 2020	Jul-Sep 2019	Oct-Dec 2018	Jan-Mar 2018	No	No	unknown
2388	4	Apr-June 2020	Oct-Dec 2019	Oct-Dec 2017	Jan-Mar 2017	No	No	unknown
2389	4	Apr-June 2020	Oct-Dec 2019	Oct-Dec 2018	Jul-Sep 2017	No	No	unknown
3944	4	Apr-June 2020	Oct-Dec 2019	Oct-Dec 2017	Jan-Feb 2017	No	No	unknown
3973	2	Apr-June 2020	Oct-Dec 2019	Jan-Mar 2019	Oct-Dec 2018	Yes	No	unknown
4584	2	Apr-June 2020	Oct-Dec 2019	Jan-Mar 2019	Jan-Mar 2018	No	No	unknown
4299	4	Apr-June 2020	Oct-Dec 2019	Oct-Dec 2018	Oct-Dec 2017	Yes	No	unknown
3862	15	Apr-June 2020	None	None	None	No	No	unknown
2350	20	Apr-June 2020	Oct-Dec 2019	unknown	unknown	Yes	No	unknown
2365	17	Apr-June 2020	Oct-Dec 2018	Oct-Dec 2016	Jul-Sep 2013	No	No	unknown
2380	15	Apr-June 2020	Oct-Dec 2019	Oct-Dec 2018	Oct-Dec 2017	No	No	unknown
2382	19	Apr-June 2020	Jul-Sep 2018	Oct-Dec 2017	Jul-Sep 2015	No	No	unknown
3856	20	Apr-June 2020	Oct-Dec 2019	Oct-Dec 2012	None	Yes	No	Influenza B positive in Jan-Mar 2020
3857	>15 years	Apr-June 2020	unknown	unknown	unknown	unknown	No	unknown
3866	18	Apr-June 2020	Oct-Dec 2018	Oct-Dec 2017	Oct-Dec 2016	Yes	Yes	unknown

Table S1. Age, serum collection date, recent vaccination history, and relevant medical history for the unvaccinated infant and individuals in the 2-5 and 15-20 year age cohorts from Seattle, Washington.

Table S2.

Study ID	Age at serum collection (in years)	Serum collection date (quarter)	Received flu vaccine in 2019-2020 season?	2019-2020 flu vaccine date (quarter)	Received flu vaccine in 2018-2019 season?
34C	45	Apr-June 2020	Yes	Oct-Dec 2019	Yes
199C	44	Apr-June 2020	Yes	Oct-Dec 2019	Yes
197C	42	Jul-Sep 2020	No	N/A	Yes
18C	43	Jul-Sep 2020	Yes	Jul-Sep 2019	Yes
33C	43	Jul-Sep 2020	Yes	Oct-Dec 2019	Yes
215C	43	Jul-Sep 2020	Yes	Oct-Dec 2019	Yes
74C	42	Oct-Dec 2020	No	N/A	No
210C	45	Oct-Dec 2020	Yes	Oct-Dec 2019	Yes
150C	43	Oct-Dec 2020	Yes	Oct-Dec 2019	Yes
68C	44	Oct-Dec 2020	No	N/A	Yes

Table S2. Age, serum collection date, and recent vaccination history for individuals in the 40-45 year age cohort from Seattle, Washington.

bioRxiv preprint doi: https://doi.org/10.1101/2023.12.12.571235; this version posted December 12, 2023. The copyright holder for this preprint (which was not certified by peer review) is the author/funder, who has granted bioRxiv a license to display the preprint in perpetuity. It is made available under aCC-BY 4.0 International license.

Table S3.

**Primers for amplifying WSN-flanked H3 HA coding sequence**	
Forward linearizing primer (primer_088)	gcaaaactactggtcctgttatatgcatttgtagc
Reverse linearizing primer (primer_089)	ctcattatatacagatgttgcatcggatgttgcc
**Primers for mutagenizing H3 HA ectodomain**	
General mutagenic primer pool (forward and reverse)	https://github.com/dms-vep/flu_h3_hk19_dms/blob/main/library_design/hk19_primers.csv
Epitope primer pool (forward and reverse)	https://github.com/dms-vep/flu_h3_hk19_dms/blob/main/library_design/hk19_single_epitope_primers.csv
Paired epitope primer pool (forward and reverse)	https://github.com/dms-vep/flu_h3_hk19_dms/blob/main/library_design/hk19_paired_epitope_primers.csv
**Primers for barcoding WSN-flanked H3 HA sequences**	
Forward linearizing primer (primer_088)	gcaaaactactggtcctgttatatgcatttgtagc
Reverse barcoding primer (primer_090)	acactctttccctacacgacgctcttccgatctNNNNNNNNNNNNNNNNctcattatatacagatgttgc
**Primers for amplifying H3 HA C-terminus with GFP overlap**	
Forward GFP overlap primer (primer_115)	TGGACGAGCTGTACAAGTAATAGgttgagctgaagtcaggatacaaagattggatc
Reverse linearizing primer (primer_089)	ctcattatatacagatgttgcatcggatgttgcc
**Primers for generating RNA spike-in template with T7 promoter**	
Forward U12-annealing primer (primer_113)	agcaaaagcaggggaaaataaaaacaacc
Reverse T7-appending primer (primer_116)	TTACGATAATACGACTCACTATAGGGagtagaaacaagggtgtttttccttatatttctg
**Primers for reverse transcription of viral barcodes**	
Viral mRNA-annealing RT primer (primer_110)	ggcaacatccgatgcaacatctgtatataatga
**Round one Illumina barcode sequencing preparation primers**	
Illumina round 1 forward primer (primer_098)	GTGACTGGAGTTCAGACGTGTGCTCTTCCGATCTggcaacatccgatgcaacatctgtatataatgag
Illumina round 1 reverse primer (primer_099)	acactctttccctacacgacgctcttccgatct

bioRxiv preprint doi: https://doi.org/10.1101/2023.12.12.571235; this version posted December 12, 2023. The copyright holder for this preprint (which was not certified by peer review) is the author/funder, who has granted bioRxiv a license to display the preprint in perpetuity. It is made available under aCC-BY 4.0 International license.

Round two Illumina barcode sequencing preparation primers	
Unique i5 indexing prime	AATGATACGGCGACCACCGAGATCTACACxxxxxxxxxxACACTCTTTCCCTACACGACGCTCTTCCGATCT<br><br>Where "xxxxxxxxxx" are NextFlex indices unique to each primer.
Unique i7 indexing primer	CAAGCAGAAGACGGCATACGAGATtatcttcagcGTGACTGGAGTTCAGACGTGTGCTCTTCCGATCT<br><br>Where "xxxxxxxxxx" are NextFlex indices unique to each primer.

Table S3. Primers and sequences referenced in methods.
